# Sociodemographic Factors Associated With Established and Novel Antenatal Vaccination Uptake in a Cohort of Pregnant Women in Uganda

**DOI:** 10.1097/INF.0000000000004644

**Published:** 2025-02-14

**Authors:** Sarah Sturrock, Hannah Davies, Gordon Rukundo, Cleophas Komugisha, Sam Kipyeko, Eve Nakabembe, Robert Mboizi, Musa Sekikubo, Kirsty Le Doare

**Affiliations:** From the * Centre for Neonatal and Paediatric Infection, City St George’s, University of London, UK; † Department of Clinical Research, Faculty of Infectious and Tropical Diseases, London School of Hygiene and Tropical Medicine, UK; ‡ Makerere University-Johns Hopkins University Research Collaboration, Uganda; § Department of Obstetrics and Gynaecology, School of Medicine, College of Health Sciences, Makerere University, Uganda.

**Keywords:** maternal vaccination, novel vaccination, SARS-CoV-2, global health

## Abstract

**Introduction:**

Vaccination is a key strategy to safeguard the health of pregnant women and newborns. Although vaccine acceptance is often higher in low- and middle-income countries, the COVID-19 pandemic has highlighted increasing vaccine hesitancy. Vaccine hesitancy, specifically in pregnant women, must be understood to increase uptake. We used data from a Ugandan pregnancy registry study to examine sociodemographic factors linked to uptake of vaccines (tetanus toxoid and later COVID-19) in pregnancy.

**Methods:**

Pregnant women were recruited in Kampala, Uganda, as part of the PREPARE (Prevention of invasive Group B Streptococcus disease in young infants: a pathway for the evaluation & licensure of an investigational maternal GBS vaccine) study from September 1, 2020 until February 24, 2022. Demographic, socioeconomic and obstetric data were collected alongside COVID-19 and tetanus vaccination.

**Results:**

One thousand five hundred sixty-eight participants were included: 151 (10%) were unvaccinated, 11 (1%) received COVID-19 vaccine only, 1230 (78%) received a tetanus vaccine only and 176 (11%) received both. Lower vaccination rates were seen in participants attending fewer than 4 antenatal care episodes (*P* < 0.001), and those with higher parity (*P* = 0.036). Higher vaccination rates were seen with a managerial or professional occupation or higher maternal education level, but paternal occupation was not significantly associated with maternal vaccination. Parish vaccination rates varied from 0% to 100%, with 49 (29%) of parishes showing a vaccination rate <90%.

**Conclusions:**

This study highlights antenatal care as a key route for health promotion, and the stark difference in uptake between new and established antenatal vaccines. Further qualitative studies should investigate effective interventions to establish the safety and benefit of newer maternal vaccines across all demographic groups.

Reducing childhood mortality is a key global health target.^1^ Although successes have been seen in reducing under-5 mortality, improvement in neonatal mortality (death in the first 28 days of life) has lagged behind.^2–4^ One reason for this is the underutilization of immunization during pregnancy as a strategy for reducing preventable neonatal deaths. Increasing vaccination coverage stands to have a monumental impact on mortality for children born today, with an estimated 72% lower mortality possible for children born in 2019 compared to what would be expected without vaccination.^5^ However, with most recommended vaccination programs starting after the first month of life and requiring multiple doses to achieve immunity, newborns are left without protection against common pathogens in this crucial vulnerable period.^6^

Vaccination during pregnancy provides a unique opportunity to protect newborns from vaccine-preventable infectious diseases via passive immunity. Vaccination programs for expectant mothers have been very effective in reducing morbidity and mortality, leading to a 96% reduction in tetanus-related neonatal deaths.^7^ These programs are of particular importance in low-resource settings where the majority of under-5 mortality occurs.^3^ New vaccines against respiratory syncytial virus and Group B Streptococcus are on the horizon and will hopefully continue to reduce the burden of these pathogens in the neonatal population.^8^

Unfortunately, programs to vaccinate during pregnancy depend on adequate uptake to have a meaningful impact on neonatal outcomes. Many factors affect vaccine coverage during pregnancy in low-resource settings (LRS), including availability of vaccine supply, infrastructure to deliver vaccines, personal knowledge of vaccine benefits, and societal views of the risks of vaccination.^9–11^ Some of these factors are common to all vaccines, whereas some may be specific to a particular vaccine.^9^ The social context is particularly important during pregnancy^10^—the influence of churches, partners and husbands have all been highlighted as important to women in low-resource settings.^12–15^ Recommendations from trusted healthcare workers and perceived safety of vaccines have also been found to be key influencing factors in pregnancy.^10,15–17^

The COVID-19 response presented an entirely new vaccine, developed at an unprecedented pace, for adoption into the maternal vaccination schedule.^18,19^ COVID-19 has been associated in multiple studies with adverse pregnancy outcomes including preeclampsia, stillbirth and premature delivery.^20,21^ These adverse outcomes were more common among pregnant women in LRS,^22^ demonstrating the importance of maximizing vaccine uptake in countries like Uganda. However, qualitative studies have demonstrated a lack of confidence in the COVID-19 vaccines in LRS.^23^ This could relate to the issues affecting all vaccines described above, and to the lack of COVID-19 vaccine trials and monitoring infrastructure in LRS.^24,25^

This study aims to quantify the uptake of established and novel vaccines during pregnancy, namely tetanus toxoid and COVID-19, in an urban LRS. We used data from a pregnancy registry study to compare uptake of the 2 vaccines from the introduction of the COVID-19 vaccine and to examine whether any demographic or social factors impacted a woman’s likelihood to take up either vaccine.

## METHODS

### Population

Participants were selected for inclusion from the PREPARE (Prevention of invasive Group B Streptococcus disease in young infants: a pathway for the evaluation & licensure of an investigational maternal GBS vaccine) study cohort. We describe below the recruitment strategy and criteria for inclusion in the overall PREPARE study, followed by details of how the cohort for this study was selected.

#### The PREPARE study

The PREPARE study recruited participants at Kawempe National Referral Hospital in Kampala, Uganda.^26^ This is a large national referral hospital caring for high-risk pregnancies referred from surrounding areas in addition to routine deliveries from the local community. The neonatal unit admits 11,000 babies per year, and there are approximately 25,000 births per year at Kawempe.^27^ Recruitment for the total PREPARE study occurred between September 1, 2020 and Februar 25, 2022.

Participants were eligible for inclusion in the PREPARE study if they were pregnant, adult or an emancipated minor ≥14 years, and attending an antenatal clinic at the study site. They needed to be able and willing to provide written informed consent. Participants were eligible if they were presenting in the first or second trimester of pregnancy as defined by abdominal ultrasound scan, and planning to attend for antenatal care and delivery at the study site. They also needed to be planning to stay within Kampala or nearby Wakiso district until the infant was at least 9 months old, be willing to attend immunization visits at 6 weeks following the end of pregnancy and end-of-follow-up visits at 14 weeks or 9 months of age, and willing to be contacted by phone and/or be visited at home.

Eligibility was assessed when participants attended the antenatal clinic, and participants were referred to the study team by their primary care provider. Participants were then included and assigned a participant identification number if they were able and willing to provide informed consent to participate in the study.^26^

#### Cohort Selection

Participants were selected from the PREPARE cohort if they had been pregnant during the period when COVID-19 vaccination was available to pregnant women at Kawempe National Referral Hospital, the study site.

COVID-19 vaccination in Uganda was initially made available to healthcare workers only, before opening to the rest of the adult population. The date at which vaccinations became available differed across health facilities due to supply inconsistencies. For this substudy, we aimed to determine as accurately as possible the time during which COVID-19 vaccination was available to pregnant women at our study site. We reviewed the vaccination data in the entire PREPARE cohort to find the first participant who received a COVID-19 vaccine at Kawempe National Hospital who was not a healthcare worker. This vaccine was administered on January 19, 2022, and so all participants from PREPARE who delivered after January 19, 2022 were included in the cohort for this study.

### Data Collection

Data were collected via a secure electronic case report form (CRF) with reference to scanned electronic medical records from the study site. The following information was then extracted from the CRFs for this study:

1. Demographic information: occupation, parish of residence, age and religion
2. COVID-19 and tetanus vaccination status: date, brand and number of doses where known
3. Admissions or attendances to hospital during pregnancy
4. Antenatal care episodes

The number of antenatal care episodes were grouped according to adherence to the current WHO recommendations of 8+ antenatal contacts and to the previous recommendations of 4+ antenatal contacts.^28,29^ Eight or more antenatal visits were classified as “adequate,” 4–7 visits as “moderate” and <4 as “insufficient.” Occupations were grouped into categories according to the International Labour Organization’s standard classification guidelines.^30^

### Statistical Analysis

Data were extracted from the online REDCap database to Microsoft Excel. Categorical variables were compared between groups using χ^2^ tests and Fisher exact tests (according to the minimum number of values in each category). Crude odds ratios for factors associated with maternal vaccine uptake were calculated, with those showing a statistically significant relationship included in a logistic regression. Christian, managerial/professional occupation and not completing primary education, were used as the reference categories for religion, maternal occupation and maternal education, respectively. The number of antenatal clinic visits was used as a continuous variable in the logistic regression. All statistical analysis was completed in R Studio.^31^ The geographical map of Uganda’s parishes was created with Datawrapper.^32^

### Ethical Approval

Ethical approval for the PREPARE study was sought from the Makerere University School of Medicine Research Ethics Committee SOMREC (#2020-089), Uganda National Council of Science and Technology (#HS623S) and St George’s School of Medicine Research Ethics Committee (#2020.0146).^26^

## RESULTS

### Receipt of Any Maternal Vaccine

From the 3423 participants recruited to the PREPARE study, a total of 1568 participants were included in this study (see Figure, Supplemental Digital Content 1, http://links.lww.com/INF/F905). The median age of included participants was 25 (IQR 22–29). Most participants were Christian (69%), with the remainder being Muslim (28%), or with no religion stated (3%, see Table, Supplemental Digital Content 2, http://links.lww.com/INF/F906 for further demographic details of participants). Half (52%) of participants for whom residential details were available lived in the Kampala district. Most participants had received at least 1 vaccine: 1230 (78%) had received a tetanus vaccine only, but only 176 (11%) received both vaccines. Fewer (n = 11, 1%) received a COVID-19 vaccine only, and 151 (10%) received neither vaccine.

Although the lowest vaccination rates were seen in women <18 years (82% vaccinated), the difference with other age groups did not reach statistical significance. Higher vaccination rates were seen among employed women (90% of those in managerial or professional occupations, 91% of those in other occupations), compared to the lower rates seen in women who were not employed outside the home (86%, *P* = 0.015). Paternal occupation was not associated with vaccination (*P* = 0.269). Lower vaccination rates were seen in women with a lower educational level (88% of those who had not completed primary school compared to 95% of those who completed secondary, and 92% with university education, *P* = 0.047). Lower vaccination rates were observed in Muslim (88%) and Protestant women (88%) than in other religious categories, but this trend was not statistically significant.

One hundred twenty-five different parishes were represented in this study, with 95% of participants falling within Kampala or the neighboring Wakiso district. Vaccination rate varied by parish, with a broad tendency for parishes further from Kampala to show a lower vaccination rate (see Figure, Supplemental Digital Content 3, http://links.lww.com/INF/F907). However, some parishes within the center of Kampala also showed lower vaccination rates; Mulago I, Kawempe II and Makerere I parishes all had vaccination rates <90% despite being within the urban center close to the study site.

Number of antenatal clinic visits was significantly associated with vaccination uptake, with 86% of unvaccinated mothers attending fewer than 4 visits (compared to 56% of vaccinated women, *P* < 0.001, see Table, Supplemental Digital Content 4, http://links.lww.com/INF/F908). Women with parity ≥5 had lower rates of vaccination than women with lower parity (79% compared to 93% of those with parity of 1 or 3, *P* = 0.036). There was no significant association with gravidity.

### COVID-19 Vaccine Uptake

Women in the older categories were more likely to have received a COVID vaccine than younger women, with 12% of women 35 and older vaccinated compared to 8% of under 18-year-olds (*P* = 0.024, see Table, Supplemental Digital Content 5, http://links.lww.com/INF/F909). Women in managerial or professional occupations were also more likely to be vaccinated than those with other occupations or those not employed (*P* = 0.029). Women with a university-level education were also more likely to be vaccinated against COVID-19 (18%) than those with primary education (11%, *P* = 0.012). No significant associations were found between maternal religion and receipt of a COVID-19 vaccine.

Receipt of a COVID-19 vaccine specifically was significantly associated with maternal parity, with higher COVID-19 vaccine uptake with increasing parity (see Table, Supplemental Digital Content 6, http://links.lww.com/INF/F910). For example, 26% of those who were vaccinated had a parity of 3 or higher, compared to 17% of those who were unvaccinated (*P* = 0.019). Mothers who received a COVID-19 vaccine were also more likely to have attended at least 4 antenatal visits (54%) than those who were not vaccinated (42%, *P* = 0.007).

### Logistic Regression

Results of logistic regression modeling can be seen in Table, Supplemental Digital Content 7, http://links.lww.com/INF/F911. In the unadjusted analysis, maternal age, religion and parity were not significantly associated with vaccination uptake. Compared to women who were not employed, those with a managerial or professional occupation were more likely to be vaccinated (crude OR, 1.79; 95% CI: 1.11–2.89). Women with a completed secondary or tertiary education were also more likely to be vaccinated than those with primary level or less education (crude OR, 2.01; 95% CI: 1.09–3.71). Women were more likely to have received a vaccine if they had attended more antenatal clinic visits (crude OR, 1.91; 95% CI: 1.71–2.14). Maternal age, occupation, education and number of antenatal visits were included in the final logistic regression model. In this model, the only factor that was significantly associated with vaccine uptake was the number of antenatal clinic visits (adjusted OR, 2.42; 95% CI: 2.05–2.85).

## DISCUSSION

We found that COVID-19 vaccine uptake was significantly lower than tetanus toxoid vaccine uptake in a cohort of pregnant women in Kampala, Uganda. We found that similar factors were associated with both COVID-19 and general vaccine uptake: maternal occupation, maternal education, previous deliveries and attendance at the antenatal clinic. Maternal age was significantly associated with COVID-19 vaccine uptake, but not with general vaccine uptake. We also found significant geographic variation in vaccine uptake; although urban parishes generally showed higher vaccination rates, some isolated parishes within urban centers had lower vaccination rates than their neighbors.

To date, as with vaccine trials themselves, there is little data quantifying actual COVID-19 vaccine uptake in pregnant populations in Africa. One study from Sudan found very low COVID-19 vaccine uptake (2.7%), but this was not compared with other vaccines and only examined women over 2 months.^33^ The minimal actual uptake in this study contrasts with the 28% of pregnant participants in a Cameroonian study and 62% in Ethiopia who said they were interested in receiving a COVID-19 vaccine.^34,35^ In Uganda, 1 study reported that 11% of women of childbearing age were vaccinated against COVID-19 and 76% were willing to be vaccinated, although this data was collected relatively early in Uganda’s pandemic (September–November 2021).^36^ The Uganda National Institute of Public Health reported that vaccine uptake in the general population reached 42% by Q2 of 2022,^37^ almost 4 times higher than in our cohort of pregnant women. Although public health messaging was given from the Ugandan Ministry of Health via billboards, radio and social media about COVID-19 and the safety of vaccination,^23^ it may be that many pregnant women specifically did not feel sufficiently convinced of the need for, or the safety of, COVID-19 vaccination to actually take up the vaccine when offered. The long-term position of tetanus toxoid as a safe and recommended vaccination in pregnancy could explain its higher uptake rate.

Higher parity was associated with COVID-19 and general vaccination uptake, though the trends were reversed between the 2 outcomes. Women with higher parity were more likely to take up a COVID-19 vaccine but less likely to have been vaccinated with either COVID-19 or tetanus vaccines. This could reflect the higher risk of severe COVID-19 in older mothers, given that increasing age was also associated with COVID-19 vaccine uptake and previous similar findings from Uganda earlier in the pandemic.^36^ For uptake of all vaccines, it is important to note that the World Health Organization recommends a maximum of 5 doses of tetanus toxoid vaccine in a lifetime. Therefore, mothers with multiple previous pregnancies may have already received sufficient tetanus toxoid in prior pregnancies. Alternatively, mothers with higher parity and few previous complications may feel the benefits of vaccination are fewer; a similar association with higher parity was found in an Ivory Coast study of tetanus toxoid and malaria preventive therapy.^38^

Attendance at antenatal clinic was positively associated both with COVID-19 vaccine uptake and general vaccination uptake. This is consistent with multiple studies in the region of maternal vaccination.^38,39^ Qualitative studies have previously shown the influence that healthcare workers can have on mothers’ decisions to be vaccinated, both by providing a trusted source of information on the benefits and reassurance about potential side effects.^10,15–17^ Although we do not know specifically what health messaging was given to mothers in this study from healthcare workers, the association between antenatal clinic attendance and vaccine uptake may suggest that this was a source of information for them. Antenatal clinics provide a pregnancy-specific point of contact with healthcare for expectant mothers and do not depend on the mother being interested in vaccination to attend. From a more logistical point of view, attending more antenatal clinic visits may demonstrate an ability to travel to the clinic either due to its geographic proximity or the mother’s resources. However, it must be noted that the length of pregnancy may affect both antenatal clinic attendance and vaccine uptake. Premature delivery could limit the opportunities available for both, and we did not examine the effect of prematurity on the relationship between antenatal clinic attendance and vaccination uptake in this study. We found that maternal occupation was also linked to COVID-19 vaccine uptake and that more rural parishes showed lower vaccination rates. Distance from a healthcare facility has been found to be linked to vaccine uptake in other studies,^39^ and may be mitigated by greater economic security and the ability to access private healthcare and transport. A desire to prevent disease has increased the chance of vaccination in qualitative studies in Uganda; attendance at antenatal clinic may be a marker of caution towards the mother’s health and that of her baby which could include a desire for disease prevention.^15^

We found that maternal level of education was significantly associated with both COVID-19 and general vaccination uptake, in agreement with other studies in similar settings.^34,35,38^ However, the association was not significant in the final logistic regression analysis, and 1 other study has found no link between education and vaccine hesitancy across multiple low- and middle-income countries.^17^ Our cohort did have a range of educational attainment, and we found the highest vaccination rate in mothers who had completed secondary education (higher even than those with university education). This could be explained by mothers without primary education having less access to healthcare and therefore health promotion messaging, either for financial or geographical regions. The vaccine hesitancy in more educated mothers may be more complex to understand; within Africa, vaccine acceptance can be affected across societal groups by perceptions of politicians promoting or criticizing vaccines, or access to online disinformation.^40^ Some vaccine hesitancy may be justifiably linked to previous unethical research practices by pharmaceutical companies in low-resource settings.^41^ Finally, vaccine hesitancy has been shown to be lower in those who have felt a personal impact of the COVID-19 pandemic.^42^ More educated members of society with better access to healthcare and remote working may have felt less personally affected by the pandemic, although equally, they may have had more to lose in terms of international travel and business.

The strength of this study is its ability to provide COVID-19 vaccine uptake data within the context of general vaccine uptake, highlighting similarities and differences in the determinants of COVID-19 vaccine uptake. We have also collected data from an observational pregnancy registry study, which allows us to study a more representative sample of pregnant women in Uganda than those specifically consenting to a vaccination-related study. Our dataset also includes women from the beginning of the COVID-19 vaccine’s introduction to almost 18 months later, reducing the impact of short-term fluctuations in vaccine availability or promotion. Quantifying vaccine uptake in a large and generalizable dataset such as this provides key information for policymakers on where efforts should be targeted to boost vaccine coverage amongst pregnant women.

However, there are limitations to our study. First, women were recruited from a maternity healthcare service. Therefore, we have not captured vaccination data from women who have had no contact with healthcare services during their pregnancy, who would be likely to have much lower vaccination rates. Additionally, the PREPARE study from which this cohort was selected used convenience sampling, so may not be representative of the population of pregnant women attending the study site and suffer from selection bias. Women from certain groups may have been more likely to participate—for example, those who are employed may be more likely to stay in the area for the required 9 months and be able to participate than those who were unemployed. Although some of our participants came from more rural areas within Uganda, most of our participants were from urban areas, limiting our ability to estimate vaccine uptake in these rural populations. Given the relative lack of access to healthcare in rural settings, these populations should not be forgotten in efforts to increase vaccine uptake if maternal and neonatal mortality is to be improved. These factors limit the generalizability of this study to the wider Ugandan population.

Vaccination was recorded either by documentation of being given at the study site, or by women’s self-reporting of vaccines given elsewhere. Therefore, we may have underestimated vaccine uptake in some women who did not recall being vaccinated at another facility. Many participants could not recall their date of vaccination, so the vaccination date was likely estimated for a significant portion of participants. Although we were informed by hospital administration that vaccination against COVID-19 was available throughout the study period, it is possible that transient stockouts may have limited access to the vaccine for some participants. Finally, our data is purely quantitative. Although it provides a useful overview of vaccine uptake in this population, we are not able to definitively make conclusions about the reasons behind vaccine uptake or refusal.

## CONCLUSION

This study highlights the stark differential uptake during pregnancy between an established vaccine (tetanus toxoid) and the novel COVID-19 vaccine. We found that antenatal care episodes are a key route for health promotion for all vaccines, whereas the sociodemographic factors associated with vaccine uptake differed between COVID-19 and tetanus. Further qualitative study should investigate the reasons behind the low uptake of the COVID-19 vaccine in women who are otherwise willing to be vaccinated and develop effective interventions to communicate the safety and benefit of newer maternal vaccines. While good coverage can be achieved in low-resource settings, much more work remains to be done to ensure future developments in vaccinology and the introduction of new vaccines during pregnancy translates to a reduction in neonatal and child mortality.

## Supplementary Material


