# Supplementary figures and images for: Sociodemographic Factors Associated With Established and Novel Antenatal Vaccination Uptake in a Cohort of Pregnant Women in Uganda
Source: Pediatr Infect Dis J. 2025 Feb 14;44(2):S92–6. doi: 10.1097/INF.0000000000004644 (PMC12178161; doi:10.1097/INF.0000000000004644)

**SUPPLEMENTAL DIGITAL CONTENT 1.** Selection of participants from the PREPARE study cohort

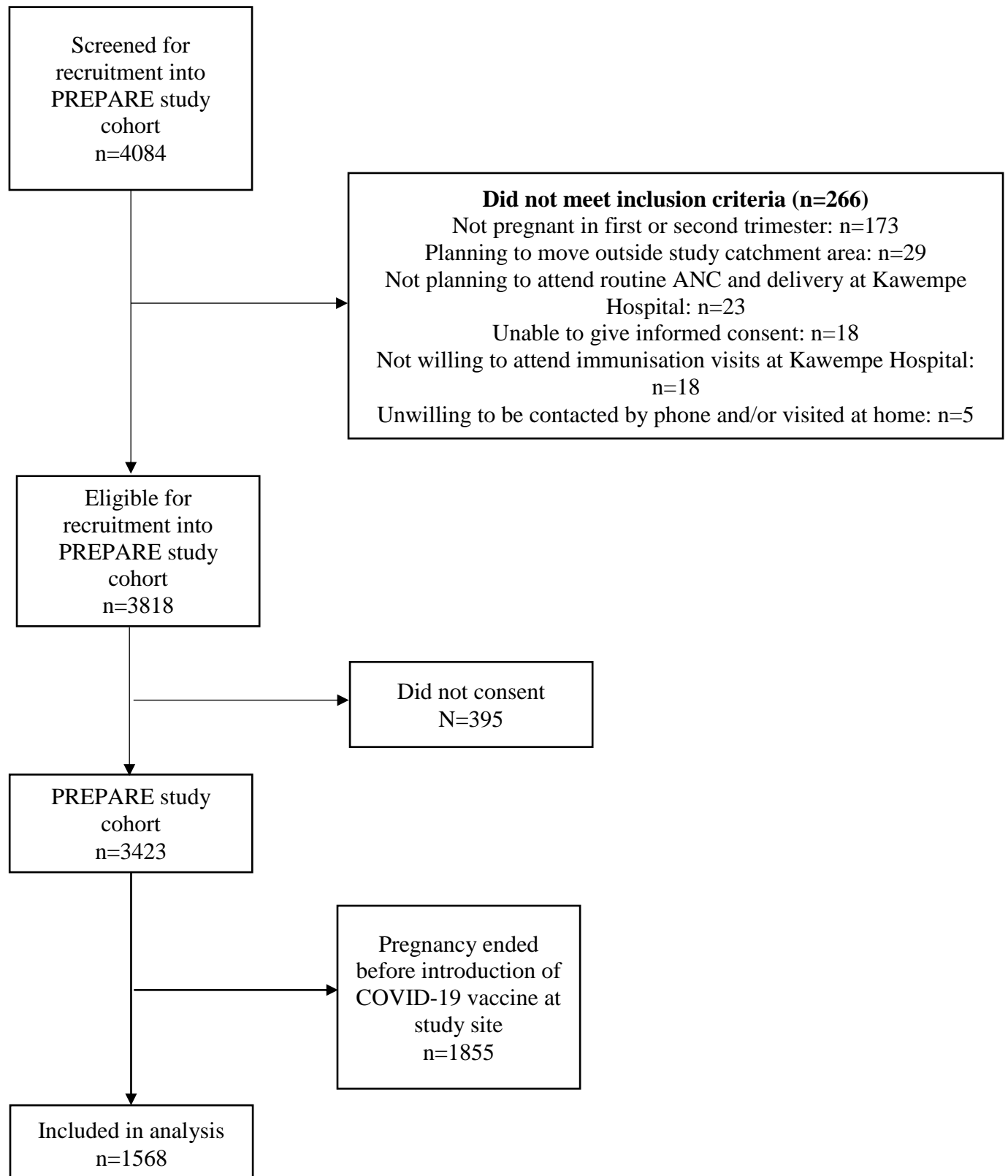

Supplement: Supplementary file 1 [file inf-44-s092-s001.pdf]
