# Supplementary material for: Sociodemographic Factors Associated With Established and Novel Antenatal Vaccination Uptake in a Cohort of Pregnant Women in Uganda
Source: Pediatr Infect Dis J. 2025 Feb 14;44(2):S92–6. doi: 10.1097/INF.0000000000004644 (PMC12178161; doi:10.1097/INF.0000000000004644)
Supplement: Supplementary file 2 [file inf-44-s092-s002.pdf]

SUPPLEMENTAL DIGITAL CONTENT 2. Factors associated with maternal vaccination status (all vaccines)

	**Total (%)**	**Vaccinated (%)**	**Unvaccinated (%)**	**p value (Chi^2)**
**All participants**	1568	1417 (90)	151 (10)	-
**Age group**				
<18	38 (2)	31 (2)	7 (5)	0.182
19-25	759 (48)	682 (48)	77 (51)	
26-34	656 (42)	601 (42)	55 (36)	
35+	115 (7)	103 (7)	12 (8)	
**Religion**				
Christian	1088 (69)	992 (70)	96 (64)	0.166*
Anglican	135 (9)	122 (9)	13 (9)	
Born Again	299 (19)	274 (19)	25 (17)	
Catholic	410 (26)	378 (27)	32 (21)	
Protestant	163 (10)	143 (10)	20 (13)	
Other	81 (5)	75 (5)	6 (4)	
Muslim	436 (28)	384 (27)	52 (34)	
None stated	44 (3)	41 (3)	3 (2)	
**Occupation (maternal)**				
Managerial/professional	869 (55)	483 (34)	34 (23)	0.015
Other employment	615 (39)	617 (44)	77 (51)	
Not employed/no occupation stated	84 (5)	317 (22)	40 (26)	
**Occupation (paternal)**				
Managerial/professional	869 (55)	784 (55)	85 (56)	0.269
Other employment	615 (39)	561 (40)	54 (36)	
Not employed/no occupation stated	84 (5)	72 (5)	12 (8)	
**Maternal education level**				
Not completed primary	152 (10)	133 (9)	19 (13)	0.047
Completed primary	963 (61)	861 (61)	102 (68)	
Completed secondary	161 (10)	153 (11)	8 (5)	
University/tertiary	292 (19)	270 (19)	22 (15)	

*Fisher's exact test
