# Supplementary material for: Sociodemographic Factors Associated With Established and Novel Antenatal Vaccination Uptake in a Cohort of Pregnant Women in Uganda
Source: Pediatr Infect Dis J. 2025 Feb 14;44(2):S92–6. doi: 10.1097/INF.0000000000004644 (PMC12178161; doi:10.1097/INF.0000000000004644)

SUPPLEMENTAL DIGITAL CONTENT 3. Rate of vaccination (received ≥1 vaccine) by parish

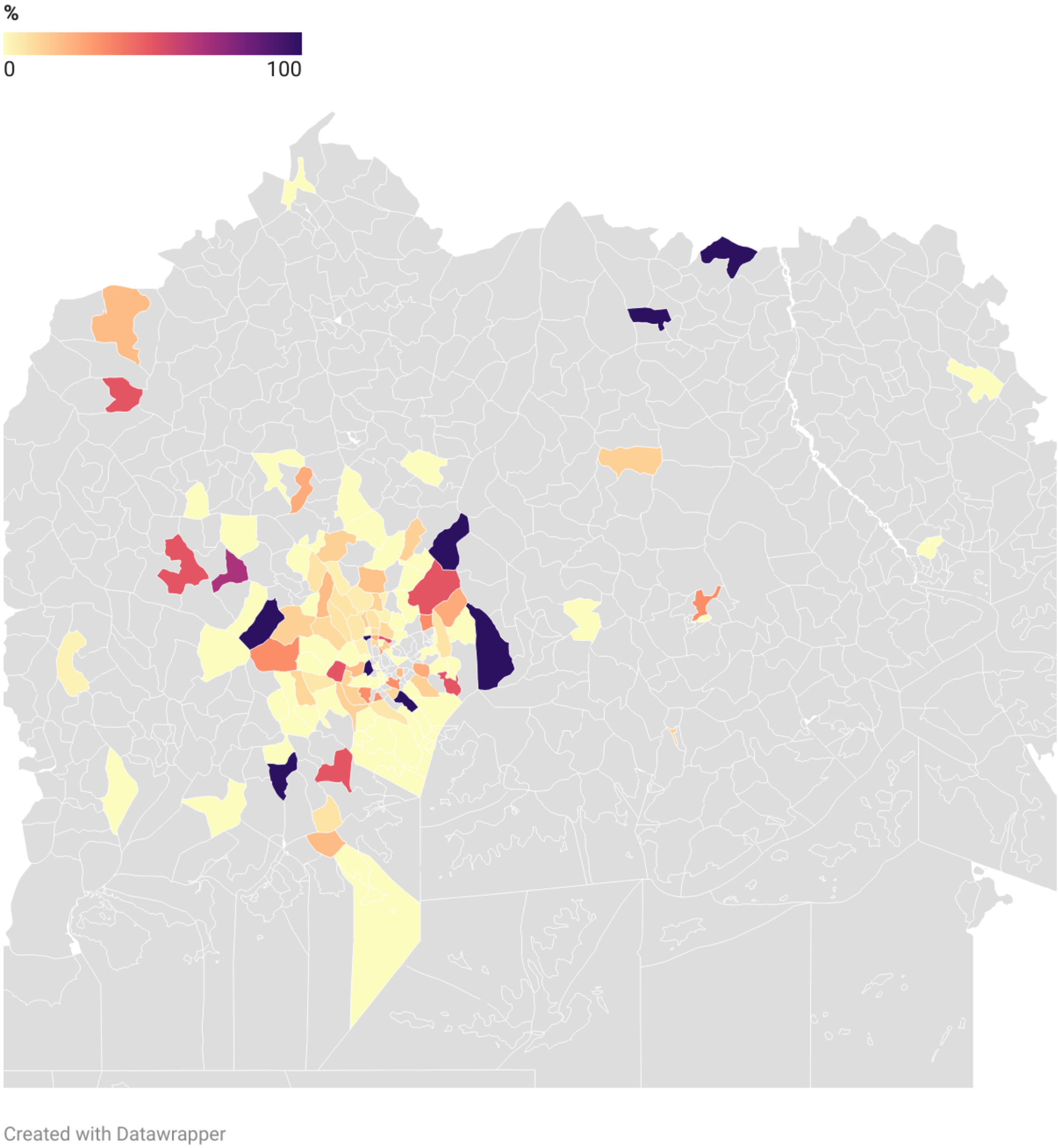

Supplement: Supplementary file 3 [file inf-44-s092-s003.pdf]
