# Supplementary material for: Sociodemographic Factors Associated With Established and Novel Antenatal Vaccination Uptake in a Cohort of Pregnant Women in Uganda
Source: Pediatr Infect Dis J. 2025 Feb 14;44(2):S92–6. doi: 10.1097/INF.0000000000004644 (PMC12178161; doi:10.1097/INF.0000000000004644)
Supplement: Supplementary file 4 [file inf-44-s092-s004.pdf]

SUPPLEMENTAL DIGITAL CONTENT 4. Obstetric factors associated with maternal vaccination (all vaccines)

		**All** N (%)	**Vaccinated** N (%)	**Unvaccinated** N (%)	**p value**
**Parity**					
	0	616 (39)	548 (39)	68 (45)	0.036**
	1	428 (27)	397 (28)	31 (21)	
	2	253 (16)	227 (16)	26 (17)	
	3	164 (10)	152 (11)	12 (8)	
	4	64 (4)	59 (4)	5 (3)	
	≥5	43 (3)	34 (2)	9 (6)	
**Gravidity**					
	1	504 (32)	445 (31)	59 (39)	0.146**
	2	419 (27)	389 (27)	30 (20)	
	3	277 (18)	250 (18)	27 (18)	
	4	182 (12)	168 (12)	14 (9)	
	≥5	186 (12)	165 (12)	21 (14)	
**Antenatal clinic visits**					
	<4	884 (56)	754 (53)	130 (86)	<0.001*
	4-7	678 (43)	658 (46)	20 (13)	
	≥8	6 (0)	5 (0)	1 (1)	

*Fisher's exact test
**Chi^2 test
