# Supplementary material for: Sociodemographic Factors Associated With Established and Novel Antenatal Vaccination Uptake in a Cohort of Pregnant Women in Uganda
Source: Pediatr Infect Dis J. 2025 Feb 14;44(2):S92–6. doi: 10.1097/INF.0000000000004644 (PMC12178161; doi:10.1097/INF.0000000000004644)
Supplement: Supplementary file 5 [file inf-44-s092-s005.pdf]

SUPPLEMENTAL DIGITAL CONTENT 5. Demographic factors associated with maternal COVID-19 vaccination uptake

	**Received COVID-19 vaccine N (%)**	**Did not receive COVID-19 vaccine N (%)**	**p value**
**Maternal age**			
<18	3 (2)	35 (3)	0.024*
19-25	73 (39)	686 (50)	
26-34	97 (52)	559 (40)	
35+	14 (7)	101 (7)	
**Religion**			
Christian	142 (76)	946 (69)	0.117*
Anglican	24 (13)	111 (8)	
Born Again	40 (21)	259 (19)	
Catholic	53 (28)	357 (26)	
Protestant	16 (9)	147 (11)	
Other	9 (5)	72 (5)	
Muslim	42 (22)	394 (29)	
None stated	3 (2)	41 (3)	
**Maternal occupation**			
Managerial/professional	78 (42)	439 (32)	0.029*
Other	73 (39)	621 (45)	
Not employed/no occupation stated	36 (19)	321 (23)	
**Paternal occupation**			
Managerial/professional	97 (52)	772 (56)	0.405*
Other	77 (41)	538 (39)	
Not employed/no occupation stated	13 (7)	71 (5)	
**Maternal education**			
Not completed primary	16 (9)	136 (10)	0.012*
Completed primary/some secondary	102 (55)	861 (62)	
Completed secondary	17 (9)	144 (10)	
University/tertiary	52 (28)	240 (17)	

*Fisher's exact test

**Chi^2 test
