# Supplementary material for: Sociodemographic Factors Associated With Established and Novel Antenatal Vaccination Uptake in a Cohort of Pregnant Women in Uganda
Source: Pediatr Infect Dis J. 2025 Feb 14;44(2):S92–6. doi: 10.1097/INF.0000000000004644 (PMC12178161; doi:10.1097/INF.0000000000004644)
Supplement: Supplementary file 6 [file inf-44-s092-s006.pdf]

SUPPLEMENTAL DIGITAL CONTENT 6. Obstetric factors associated with maternal COVID-19 vaccination uptake

	**Received COVID-19 vaccine N (%)**	**Did not receive COVID-19 vaccine N (%)**	**p value**
**Parity**			
0	66 (35)	550 (40)	0.019**
1	50 (27)	378 (27)	
2	23 (12)	230 (17)	
3	32 (17)	132 (10)	
4	11 (6)	53 (4)	
≥5	5 (3)	38 (3)	
**Gravidity**			
1	54 (29)	450 (33)	0.117**
2	48 (26)	371 (27)	
3	27 (14)	250 (18)	
4	30 (16)	152 (11)	
≥5	28 (15)	158 (11)	
**Antenatal clinic visits**			
<4	86 (46)	798 (58)	0.007*
4-7	100 (53)	578 (42)	
≥8	1 (1)	5 (0)	

*Fisher's exact test
**Chi^2 test
