# Supplementary material for: Sociodemographic Factors Associated With Established and Novel Antenatal Vaccination Uptake in a Cohort of Pregnant Women in Uganda
Source: Pediatr Infect Dis J. 2025 Feb 14;44(2):S92–6. doi: 10.1097/INF.0000000000004644 (PMC12178161; doi:10.1097/INF.0000000000004644)
Supplement: Supplementary file 7 [file inf-44-s092-s007.pdf]

SUPPLEMENTAL DIGITAL CONTENT 7. Logistic regression results for uptake of any maternal vaccine

	**[1417/1568]**	
	**Unadj.OR (95%CI)**	**Adj.OR (95%CI)**
**Maternal Factors**	***n*=1568**	***n*=1568**
**Maternal Age, y**	1.03 (0.99-1.06)	**0.99 (0.96-1.03)**
**Religion**		
**Christian**	1.0	…
**Muslim**	0.71 (0.50-1.02)	
**None / missing**	1.32 (0.40-4.35)	
**Maternal Occupation**		
**None**	1.0	1.0
**Managerial/professional**	1.79 (1.11-2.89)	1.52 (0.90-2.57)
**Non managerial/professional**	1.01 (0.67-1.52)	0.91 (0.59-1.40)
**Maternal education level**		
**None or some primary**	1.0	**1.0**
**Some secondary**	1.21 (0.71-2.03)	1.35 (0.77-2.38)
**Completed secondary / tertiary**	2.01 (1.09-3.71)	1.88 (0.98-3.61)
**Number of antenatal visits**	1.91 (1.71-2.14)	2.42 (2.05-2.85)
**Parity**		
**0**	1.0	
**1-3**	1.40 (0.98-1.99)	…
**>3**	0.82 (0.45-1.53)	
